# Impact of Cooling Strategies on Transfusion Requirements in Aortic Hemiarch Surgery

**DOI:** 10.1055/a-2693-4175

**Published:** 2025-09-17

**Authors:** Anthony V. Norman, Sanjana Challa, Genevieve Lyons, Alexander M. Wisniewski, Raymond J. Strobel, Michael Mazzeffi, Mark Joseph, Daniel Tang, Ramesh Singh, Michael C. Kontos, Mohammed Quader, Kenan Yount, Nicholas R. Teman, Ourania Preventza, Jared P. Beller

**Affiliations:** 1 Division of Cardiothoracic Surgery, University of Virginia, Charlottesville, Virginia; 2 Department of Public Health Sciences, University of Virginia, Charlottesville, Virginia; 3 Department of Anesthesia, University of Virginia, Charlottesville, Virginia; 4 Department of Cardiothoracic Surgery, Carilion Clinic, Roanoke, Virginia; 5 Department of Cardiothoracic Surgery, INOVA Heart and Vascular Institute, Falls Church, Virginia; 6 Department of Cardiac Surgery, Virginia Commonwealth University, Richmond, Virginia; 7 Division of Cardiothoracic Surgery, University of Colorado School of Medicine, Aurora, Colorado

**Keywords:** hypothermia, circulatory, blood, transfusion, aorta

## Abstract

**Background:**

Deep hypothermic circulatory arrest (DHCA) is associated with coagulopathy but facilitates aortic arch surgery. Conflicting data suggest moderate hypothermic circulatory arrest (MHCA) may reduce transfusion requirements. We hypothesized MHCA would reduce transfusion requirements.

**Methods:**

We studied patients undergoing aortic hemiarch surgery for nondissected, aneurysmal disease from July 2014 to May 2023 utilizing a multicenter collaborative. Patients were stratified by DHCA (14.1–20°C) and MHCA (20.1–28°C). Packed red blood cells (pRBC), fresh frozen plasma (FFP), cryoprecipitate, and platelet transfusion requirements were assessed. A negative binomial model accounting for hospital random effect was fitted to identify risk factors for increased transfusion requirements.

**Results:**

Of the 451 patients undergoing hemiarch surgery, 373 (83%) had MHCA and 78 (17%) had DHCA. MHCA patients had shorter cardiopulmonary bypass (135 minutes [105, 182] vs. 216 minutes [183, 263], *p* < 0.001) and circulatory arrest times (12 minutes [8, 17] vs. 21 minutes [16, 34], *p* < 0.001). MHCA patients received fewer pRBC (0 [0, 1] vs. 1 [0, 3], *p* < 0.001), FFP (0 [0, 3] vs. 2 [0, 4], *p* = 0.003), cryoprecipitate (1 [0, 1] vs. 1 [0, 2], *p* = 0.045), and platelet transfusions (0 [0, 1] vs. 2 [0, 2], *p* < 0.001). Unadjusted operative mortality was lower in the MHCA group (1.9 vs. 7.7%, *p* < 0.01). After risk adjustment, MHCA was associated with reduced FFP transfusion requirements (β = −0.48, SE = 0.2, *p* = 0.017). Increasing bypass time per minute was associated with increased pRBC (β = +0.01, 95% CI = 0.006–0.013, *p* < 0.001), FFP (β = +0.006, 95% CI = 0.004–0.009, *p* < 0.001), cryoprecipitate (β = +0.008, 95% CI = 0.005–0.01, *p* < 0.001), and platelet transfusions (β = +0.009, 95% CI = 0.006–0.011, *p* < 0.001).

**Conclusion:**

MHCA was associated with decreased mortality and FFP transfusions in aortic hemiarch repair. MHCA may mitigate transfusion needs via shorter cardiopulmonary bypass time compared with DHCA.

Deep hypothermic circulatory arrest (DHCA) has been utilized for nearly 50 years to facilitate a bloodless field for open aortic arch surgery. 1 Furthermore, it promotes end-organ protection resulting in a 6 to 7% decrease in cerebral oxygen consumption per 1°C decline. 2 However, DHCA precipitates physiological disarray, among which coagulopathy and bleeding are frequently cited. 3 4 5 Transfusion of blood products is not benign and associated with increased morbidity, mortality, and cost. 6 7 There also appears to be a dose-dependent effect, as greater transfusion requirements are associated with longer intensive care unit (ICU) length of stay (LOS) as well as higher rates of prolonged postoperative ventilation, renal failure, infection, and ischemic events. 7 8 Given this, moderate hypothermic circulatory arrest (MHCA) was proposed as an alternative to reduce the negative consequences of DHCA.

The optimal degree of hypothermia to minimize morbidity while providing adequate organ protection is highly debated. 3 9 10 MHCA has been shown to have potential benefits in pulmonary recovery, stroke risk, renal dysfunction, and mortality. 9 11 12 13 It also shortens cardiopulmonary bypass (CPB) times, which has otherwise been associated with increased transfusions. 14 However, when examining the impact the degree of hypothermia has on transfusion requirements, the data are less clear. Conflicting data from single-center studies have suggested MHCA may or may not reduce transfusion requirements. 3 4 15

Given the discordance of prior studies, we sought to use data from a multicenter collaborative to compare the impact of DHCA and MHCA on transfusion requirements. We hypothesized MHCA would reduce transfusion requirements and improve surgical outcomes.

## Materials and Methods

### Patient Data

The Virginia Cardiac Services Quality Initiative (VCSQI) is a collaborative of 17 hospitals in Virginia capturing 99% of all adult cardiac surgeries in the region. As patient records were deidentified, the University of Virginia Institutional Review Board exempted this study from review (Protocol #23305, deemed exempt July 14, 2021). Patient demographic and outcomes data were obtained from the STS database using a sample including adult patients who underwent aortic hemiarch surgery in the VCSQI. We excluded patients with a temperature nadir ≤14.1 or >28°C, missing transfusion data, aortic dissection, or who underwent repair with techniques other than hemiarch repair. Those with concomitant root replacement were included. MHCA was defined as temperature nadir range of 20.1 to 28°C and DHCA as a temperature nadir range of 14.1 to 20°C. The lowest temperature recorded was used.

Outcomes measured included STS operative mortality and the number of intraoperative as well as total intra- and postoperative transfusions for each of the following blood products: packed red blood cells (pRBCs), fresh frozen plasma (FFP), cryoprecipitate, and platelet units. Regarding platelet transfusions, data are only shown for those who received platelet units rather than dose packs. Platelet dose packs contain varying numbers of platelet units and may not accurately represent platelet transfusion requirements across the patient cohort.

### Statistical Analysis

Continuous variables are presented as a mean (standard deviation) for normally distributed data or a median (Q1, Q3) for non-normally distributed data. Differences in continuous variables with skewed distributions were compared using Wilcoxon rank-sum tests and normally distributed variables by Student's *t* -test. Categorical variables are presented as n (%) and compared by chi-squared or Fischer's exact tests. Missing data were imputed for variables with less than 5% missing data using the median for continuous variables and mode for categorical variables. Variables selected for the risk-adjusted analysis were deemed risk factors for bleeding or coagulopathy or were statistically different between groups. A negative binomial mixed effect model accounting for hospital as a random effect was fitted for each of the four blood product types to assess for associations between pre- and intraoperative factors and transfusion requirements. Covariates in the model included cooling strategy, age, sex, body mass index (BMI), peripheral arterial disease, adenosine-diphosphate inhibitor use, aspirin use, warfarin use, platelet count, International Normalized Ratio (INR), hematocrit, elective status, CPB time in minutes, circulatory arrest time in minutes, use of cerebral perfusion, concomitant root procedure, and year of surgery. Sensitivity and effect modification analyses were performed to assess the impact of temperature nadir as a continuous variable and for interactions between temperature and CPB and circulatory arrest times, respectively. A generalized estimation effect (GEE) model using fixed effects was used to visualize the relationship between increasing CPB time and transfusion requirements between the MHCA and DHCA groups. SAS version 9.4 (SAS Institute, Cary, NC) was used for the analysis. A cutoff of less than 0.05 for *p* -values was used for statistical significance.

## Results

### Baseline Characteristics

**Fig. 1:**
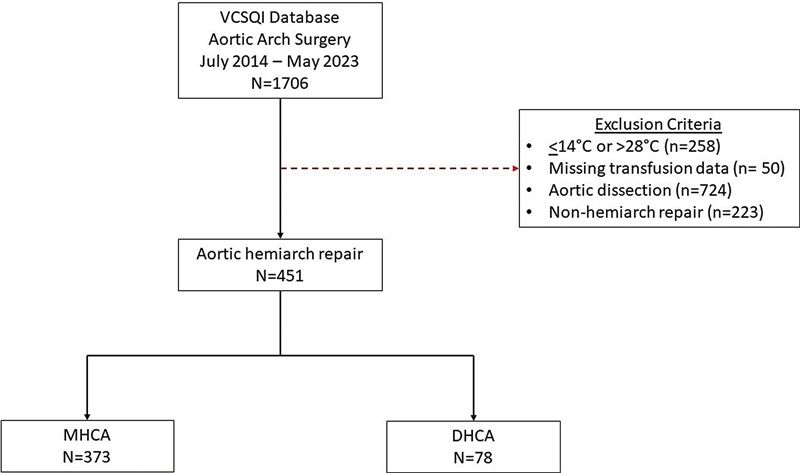
Consort diagram.

**Fig. 2:**
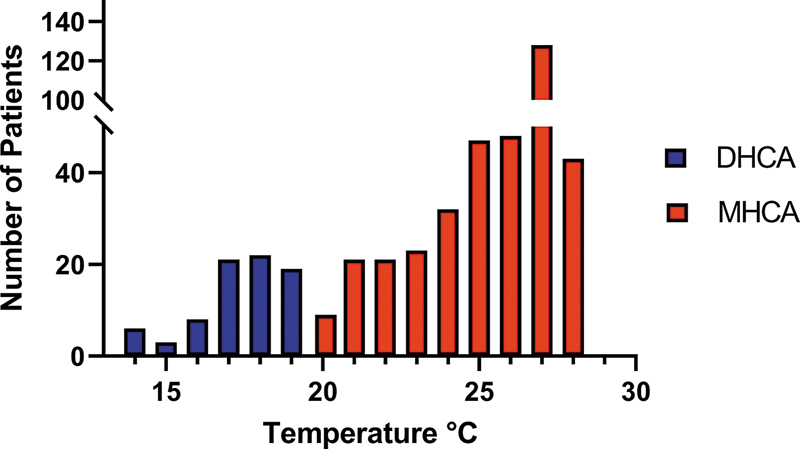
Distribution of the temperature Nadirs.

A total of 1,706 patients underwent aortic arch surgery from July 2014 through May 2023 at participating VCSQI centers. Among these, 451 patients fulfilled the inclusion criteria ( Fig. 1 ), with 373 patients in the MHCA group and 78 patients in the DHCA group. The distribution of temperature nadirs are displayed in Fig. 2 . The groups had similar baseline characteristics ( Table 1 ) including age (62 vs. 63 years, *p* = 0.922), female sex (27.1 vs. 37.2%, *p* = 0.073), White race (81 vs. 82.1%, *p* = 0.836), preoperative hematocrit (41.4 vs. 41.2%, *p* = 0.686), INR (1 vs. 1, *p* = 0.132), platelet count (224 × 10 ^9^ /L vs. 231 × 10 ^9^ /L, *p* = 0.458), preoperative heparin use (12.9 vs. 12.8%, *p* = 0.991), and elective status (85.8 vs. 79.5%, *p* = 0.16). However, the MHCA group had shorter median CPB (135 vs. 216 minutes, *p* < 0.001) and total circulatory arrest times (12 vs. 21 minutes, *p* < 0.001). They also had higher utilization of any cerebral perfusion (97.8 vs. 74.4%, *p* < 0.001) as well as antegrade cerebral perfusion (91.2 vs. 72.4%, *p* < 0.001).

**Table 1:** Baseline and intraoperative characteristics of moderate and deep hypothermic circulatory arrest patients

Variable	MHCA ( *n* = 373)	DHCA ( *n* = 78)	*p* -Value
Age	62 ± 12	63 ± 12	0.922
Female	101 (27.1)	29 (37.2)	0.073
White race	295 (81)	64 (82.1)	0.836
Elective status	320 (85.8)	62 (79.5)	0.16
BMI (kg/m ^2^ )	28 (25, 32)	29 (26, 33)	0.158
Cerebrovascular disease	47 (12.7)	12 (15.4)	0.529
Diabetes	46 (12.3)	12 (15.4)	0.464
Hypertension	298 (80.1)	64 (82.1)	0.694
Peripheral arterial disease	28 (7.51)	13 (16.7)	0.011
Prior myocardial infarction	18 (4.83)	6 (7.69)	0.297
Chronic obstructive pulmonary disease	108 (29)	24 (30.8)	0.749
Previous cardiac intervention	83 (22.3)	17 (21.8)	0.93
Previous CABG	6 (7.23)	0 (0)	0.253
Previous valve surgery	29 (34.9)	5 (29.4)	0.661
Previous other cardiac intervention	53 (63.9)	13 (76.5)	0.317
Liver disease	18 (4.84)	2 (2.56)	0.376
Preoperative dialysis dependency	2 (0.54)	0 (0)	0.516
Endocarditis	11 (2.95)	1 (1.28)	0.405
ADP inhibitor within 5 d	7 (1.88)	1 (1.28)	0.716
Anticoagulation (heparin) within 48 h	48 (12.9)	10 (12.8)	0.991
Aspirin within 5 d	175 (47.2)	37 (47.4)	0.966
Preoperative Warfarin	3 (0.8)	1 (1.28)	0.682
Preoperative hematocrit	41.4 ± 5.2	41.2 ± 4.1	0.686
Preoperative INR	1 (1, 1.1)	1 (0.99, 1.1)	0.132
Preoperative creatinine (10 9 /L)	1.03 ± 0.4	0.96 ± 0.3	0.052
Preoperative platelet count (1,000/µL)	224 ± 65	231 ± 81	0.458
Intraoperative characteristics			
Cardiopulmonary bypass time (min)	135 (105, 182)	216 (183, 263)	<0.001
Total circulatory arrest time (min)	12 (9, 19)	21 (16, 34)	<0.001
Cerebral perfusion time (min)	12 (8, 17)	24 (15, 30)	<0.001
Aortic root operation	115 (30.8)	32 (41)	0.081
Intraoperative clotting factor	73 (19.7)	15 (19.2)	0.928
Circulatory arrest with cerebral perfusion	363 (97.8)	58 (74.4)	<0.001
Antegrade cerebral perfusion (ACP)	331 (91.2)	42 (72.4)	<0.001
Retrograde cerebral perfusion (RCP)	25 (6.89)	16 (27.6)	<0.001
ACP + RCP	5 (1.38)	0 (0)	0.369

Abbreviations: ADP, adenosine diphosphate; BMI, body mass index; CABG, coronary artery bypass grafting; INR, International Normalized Ratio.

### Postoperative Results

**Fig. 3:**
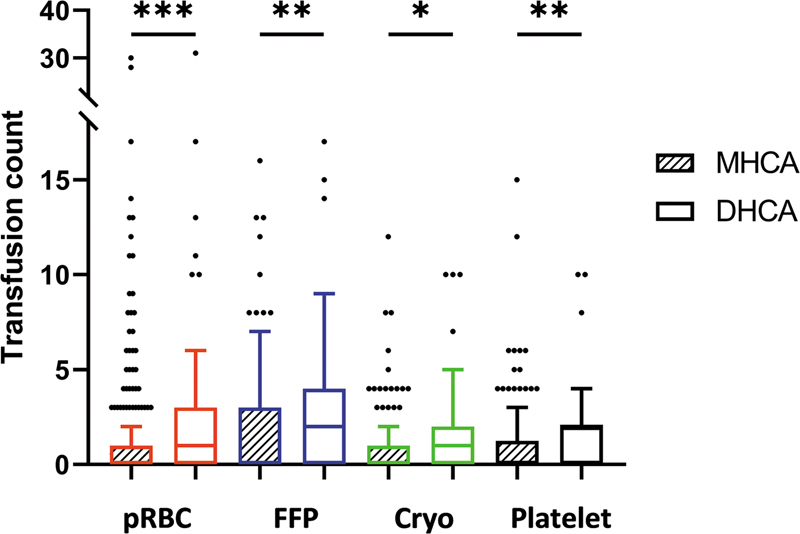
Univariate comparison of transfusion requirements by cooling strategy.

Postoperative results are detailed in Table 2 . Median total transfusion requirements were lower in the MHCA group for pRBCs (0 vs. 1, *p* < 0.001), FFP (0 vs. 2, *p* = 0.003), cryoprecipitate (0 vs. 1, *p* = 0.045), and platelets (0 vs. 2, *p* < 0.001; Fig. 3 ). The MHCA group also had lower rates of high (≥4 units) pRBC (8.06 vs. 23.1%, *p* < 0.001), cryoprecipitate (3.49 vs. 12.8%, *p* = 0.001), and platelet transfusion events (7.08 vs. 15.3%, *p* = 0.048). Operative mortality was lower in the MHCA group (1.88 vs. 7.69%, *p* = 0.005). Prolonged postoperative ventilation (10.7 vs. 20.5%, *p* = 0.017), pneumonia (1.88 vs. 12.8%, *p* < 0.001), and ICU (50 vs. 96 hours, *p* < 0.001) and hospital LOS (7 vs. 9 days, *p* < 0.001) were also lower in the MHCA group. Complications including reoperation for bleeding (2.41 vs. 2.56%, *p* = 0.937), stroke (1.61 vs. 2.56%, *p* = 0.563), and renal failure (2.96 vs. 6.41%, *p* = 0.134) were comparable.

**Table 2:** Postoperative outcomes in patients undergoing moderate versus deep hypothermic circulatory arrest

Variable	MHCA ( *n* = 373)	DHCA ( *n* = 78)	*p* -Value
Any blood product	249 (66.8)	66 (84.6)	0.002
Total packed red blood cells	0 (0, 1)	1 (0, 3)	<0.001
Total fresh frozen plasma	0 (0, 3)	2 (0, 4)	0.003
Total cryoprecipitate	0 (0, 1)	1 (0, 2)	0.045
Total platelet units	0 (0, 1)	2 (0, 2)	<0.001
≥4 units pRBC transfusion	30 (8.06)	18 (23.1)	<0.001
≥4 units FFP transfusion	84 (22.5)	24 (30.8)	0.121
≥4 units cryoprecipitate transfusion	13 (3.49)	10 (12.8)	0.001
≥4 units platelet transfusion	16 (7.08)	9 (15.3)	0.048
Intraoperative packed red blood cells	0 (0, 0)	0 (0, 2)	0.001
Intraoperative fresh frozen plasma	0 (0, 2)	2 (0, 4)	0.002
Intraoperative cryoprecipitate	0 (0, 1)	0 (0, 2)	0.096
Intraoperative platelet units	0 (0, 1)	2 (0, 2)	<0.001
Reoperation for bleeding	9 (2.41)	2 (2.56)	0.937
Delayed chest closure	13 (3.49)	6 (7.69)	0.093
Cerebral vascular accident	6 (1.61)	2 (2.56)	0.563
Renal failure	11 (2.96)	5 (6.41)	0.134
Prolonged postoperative ventilation	40 (10.7)	16 (20.5)	0.017
Atrial fibrillation	97 (26)	25 (32.1)	0.274
Pneumonia	7 (1.88)	10 (12.8)	<0.001
Sepsis	1 (0.27)	1 (1.28)	0.22
Deep sternal wound infection	1 (0.27)	0 (0)	0.645
Surgical site infection	7 (1.88)	0 (0)	0.223
Venous thromboembolism	11 (2.95)	3 (3.85)	0.679
Total ICU LOS (h)	50 (27, 94)	96 (49, 155)	<0.001
Total postoperative ventilation hours	5 (4, 9)	12 (5, 22)	<0.001
Hospital length of stay (d)	7 (4, 10)	9 (7, 17)	<0.001
Operative Mortality	7 (1.88)	6 (7.69)	0.005

Abbreviations: pRBC, packed red blood cell; FFP, fresh frozen plasma; ICU, intensive care unit; LOS, length of stay.

### Multivariable Analysis

In the multivariable analysis, MHCA was associated with decreased FFP transfusions (−0.483 units, 95% CI = −0.88 to −0.087, *p* = 0.017), but not with pRBCs (−0.177 units, 95% CI = −0.66 to 0.305, *p* = 0.47), cryoprecipitate (−0.007 units, 95% CI = −0.362 to 0.348, *p* = 0.969), or platelet transfusions requirements (−0.289 units, 95% CI = −0.748 to 0.17, *p* = 0.215). Longer CPB time was associated with increased transfusion of all blood products (pRBCs [+0.01 units/min, 95% CI = 0.006–0.013, *p* < 0.001], FFP [+0.006 units/min, 95% CI = 0.004–0.009, *p* < 0.001], cryoprecipitate [+0.008 units/min, 95% CI = 0.005–0.01, *p* < 0.001], and platelets [+0.009 units/min, 95% CI = 0.006–0.011, *p* < 0.001]; Table 3 ). Concomitant root operation was only associated with increased platelet transfusions (+0.573 units, 95% CI = 0.204–0.943, *p* = 0.003). Older age (+0.018, 95% CI = 0.003–0.034, *p* = 0.022) and female sex (+0.531, 95% CI = 0.161–0.902, *p* = 0.005) were associated with increased pRBC transfusions. BMI was also independently associated with pRBC (−0.052, 95% CI = −0.083 to −0.021, *p* = 0.001) and platelet transfusions (−0.045, 95% CI = −0.075 to −0.016, *p* = 0.003). Higher hematocrit levels were associated with decreased pRBC (−0.099, 95% CI = −0.0135 to −0.064, *p* < 0.001) and FFP (−0.036, 95% CI = −0.064 to −0.009, *p* = 0.011). There also appeared to be more FFP transfusions between 2014 and 2018 (+0.462, 95% CI = 0.127–0.797, *p* = 0.007).

**Table 3:** Impact of moderate hypothermia and cardiopulmonary bypass time on transfusion requirements in the mixed-effect model analysis

Covariate	Estimate	95% CI	Standard error	*p* -Value
Red blood cell				
MHCA	−0.177	−0.66 to 0.305	0.245	0.47
CPB time	0.01	0.006–0.013	0.002	<0.001
Fresh frozen plasma				
MHCA	−0.483	−0.88 to −0.087	0.202	0.017
CPB time	0.006	0.004–0.009	0.001	<0.001
Cryoprecipitate				
MHCA	−0.007	−0.362 to 0.348	0.18	0.969
CPB time	0.008	0.005–0.01	0.001	<0.001
Platelets				
MHCA	−0.289	−0.748 to 0.17	0.233	0.215
CPB time	0.009	0.006–0.011	0.001	<0.001

Abbreviations: CI, confidence interval; CPB, cardiopulmonary bypass; MHCA, moderate hypothermic circulatory arrest.

**Fig. 4:**
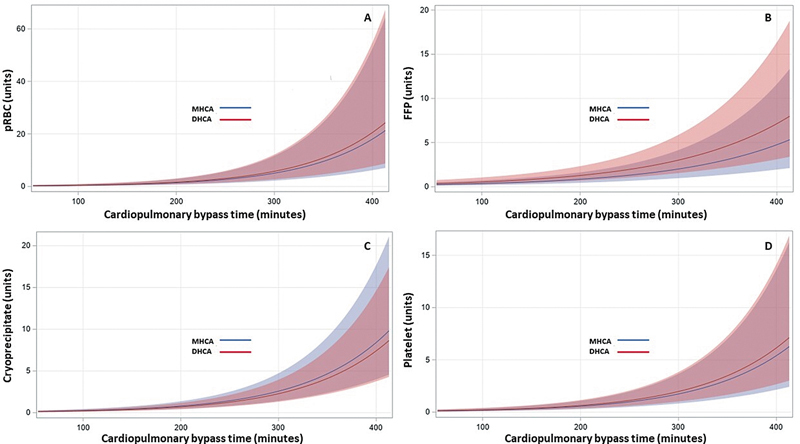
Slicefit effect plot from the multivariable GEE model illustrating the impact of increasing CPB time (mins) on transfusion requirements (units) by cooling strategy. ( **A** ) pRBCs, ( **B** ) FFP, ( **C** ) cryoprecipitate, and ( **D** ) platelets. The top 1% of CPB times were excluded to aid with visualization. CPB, cardiopulmonary bypass; GEE, generalized estimation effect; FFP, fresh frozen plasma; pRBC, packed red blood cell.

**Fig. 5:**
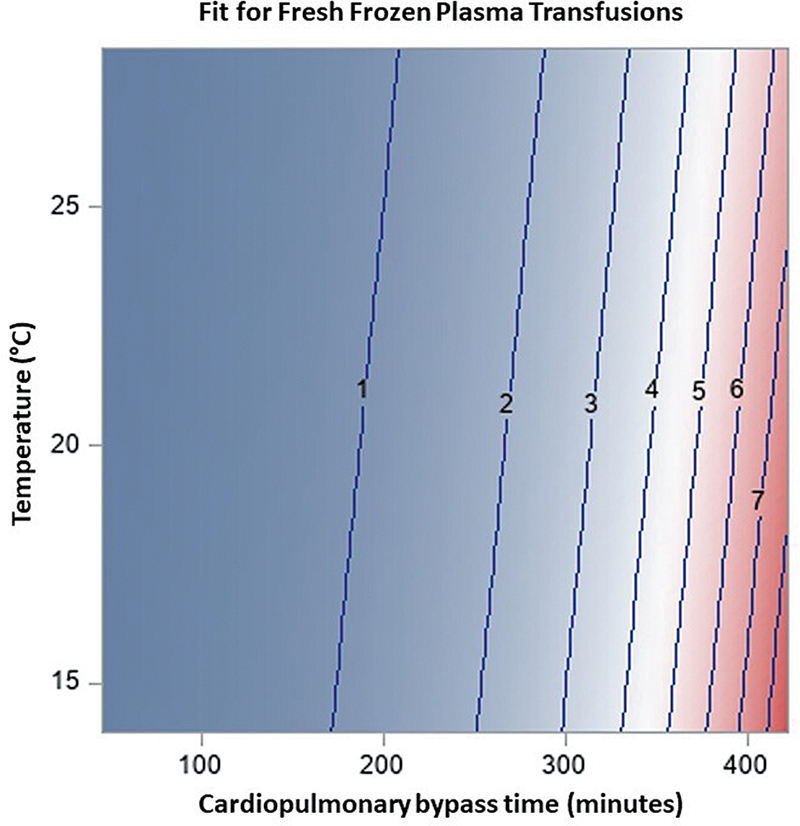
Contour plot of the GEE model demonstrating the relationship between temperature nadir (°C), cardiopulmonary bypass time (minutes), and number of fresh frozen plasma transfusions. The top 1% of CPB times were excluded to aid with visualization. CPB, cardiopulmonary bypass; GEE, generalized estimation effect.

In the sensitivity analysis, higher temperature nadirs were associated with fewer platelet transfusions (−0.06 units/°C, 95% CI = −0.116 to −0.005, *p* = 0.033). In the effect modification analysis, there was a small, significant interaction between temperature nadir as a continuous variable and both CPB (−0.0006, 95% CI = −0.001 to 0, *p* = 0.049) and circulatory arrest time (−0.006, 95% CI = −0.01 to −0.003, *p* < 0.001) regarding FFP transfusion requirements. The relationship between CPB time and transfusion requirements across MHCA and DHCA from the GEE model is illustrated in Fig. 4 . A contour plot illustrating the relationship between temperature nadir, CPB time, and FFP requirements from the model is illustrated in Fig. 5 .

## Discussion

In this multicenter study, we examined the impact of MHCA and DHCA on transfusion requirements across four blood product types for patients who underwent aortic hemiarch repair for nondissected, aneurysmal disease. Median transfusion requirements across all blood product types were lower in the MHCA group. However, MHCA was only associated with a decrease in FFP transfusions in the multivariable analysis, whereas shorter CPB time was associated with decreased transfusion of all four blood products. Nevertheless, MHCA patients had a shorter unadjusted ICU and hospital LOS as well as lower rates of prolonged postoperative ventilation, pneumonia, and operative mortality.

In aortic arch surgery, there has been a trend toward utilizing less extreme cooling temperatures for hypothermic circulatory arrest (HCA). Guidelines define DHCA as a temperature nadir range of 14.1 to 20°C and MHCA as 20.1 to 28°C. 16 Several series favor high–moderate HCA, or 24.1 to 28°C, as was seen in the distribution in our study. 9 11 This is not surprising as deeper hypothermia is associated with renal dysfunction, stroke, and infection as well as coagulopathy and increased bleeding risk. 11 12 13 15 Hypothermia has been documented to decrease coagulation cascade enzyme activity, enhance fibrinolytic activity, and induce platelet dysfunction. 5 17 18 However, how this translates clinically to transfusion requirements remains surprisingly unclear.

To the best of our knowledge, only a few single-center studies have directly compared transfusion requirements by HCA strategy. Vallabhajosyula and colleagues saw that DHCA patients were almost twice as likely to receive blood products compared with MHCA. 4 Keenan and colleagues reported modest differences with DHCA patients requiring one additional unit of plasma compared with MHCA. 3 Conversely, Tsai and colleagues saw no differences. 15 These largely negative to mildly significant findings are consistent with our results indicating MHCA itself may not have a large, clinically relevant impact on transfusion requirements. MHCA was associated with a decrease in 0.483 units of FFP. However, transfusions are not practically given in fractions. In our sensitivity analysis, higher temperature nadirs were associated with fewer platelet transfusions at a rate of −0.06 units/°C. However, the range of temperatures studied spanned only 15 degrees, which may have limited its impact on platelet transfusion requirements. In a recent meta-analysis, differences in bleeding were only appreciated between mild and deep hypothermia, but not between moderate and deep. 19 While that analysis broadly examined bleeding, it correlates with our findings in that larger temperature differences may be needed to better appreciate differences in bleeding-related outcomes. While the degree of hypothermia has been heavily debated, the duration of hypothermia must be considered.

Mazzeffi and colleagues demonstrated that DHCA and its duration contribute to increased transfusion requirements. Furthermore, they found a significant interaction between longer CPB times and longer periods under DHCA on pRBC transfusions in their effect modification analysis. 20 In contrast, we found total circulatory arrest time was not independently associated with any of the four blood products, yet CPB time was. Furthermore, there was only a modest interaction between temperature nadir and circulatory arrest time on FFP requirements. In our study, MHCA and DHCA had median circulatory arrest times of 12 and 21 minutes, respectively. This is relatively shorter compared with other published series 3 10 15 and may explain why the relationship described by Mazzeffi et al. was not appreciated. Nevertheless, they highlight the importance of CPB time, which likely plays a greater role.

The use of CPB leads to hemodilution, consumptive coagulopathy, platelet dysfunction, and cytokine upregulation. 21 Longer CPB times are associated with increased transfusion requirements. 14 CPB time was the only covariate associated with increased transfusion requirements for all four blood products in our study. MHCA patients had a median CPB time 81 minutes shorter than DHCA patients. These differences may reflect longer cooling and rewarming times in DHCA. These additional 81 minutes were likely the greatest contributor to differences in transfusion requirements. For reference, a recent meta-analysis showed that CPB times can differ by up to 97 minutes for patients undergoing DHCA versus MHCA. 19 There was also a significant interaction between temperature nadir and CPB time in the FFP model. However, based on the slicefit and contour plots, it is possible we may only start to clinically appreciate this at CPB times greater than 250 to 300 minutes. Most hemiarch repairs can be performed within that timeframe with either cooling strategy. However, anticipated operative complexity may also influence which cooling strategy is selected.

Extensive aortic operations may require longer circulatory arrest and CPB times. We limited our study to hemiarch operations to reduce the impact extensive repairs may have on transfusion requirements. Total arch repairs may incur almost double the risk of requiring transfusions compared with hemiarch repairs. 22 We did include patients undergoing concurrent aortic root operations, given discordant findings of adding a root operation on transfusion requirements and safety. 22 23 In our study, concomitant root operation increased platelet transfusion requirements but did not affect the other blood product types.

Most patients undergoing aortic hemiarch repair do so for aneurysmal disease or acute aortic dissection. Comparisons between MHCA and DHCA frequently include both, including the aforementioned studies examining transfusion requirements in arch surgery. 3 4 15 Blood contacting the nonendothelialized false lumen of an aortic dissection can activate the coagulation cascade and precipitate coagulopathy prior to surgery. 24 Our study exclusively looked at nondissected, aneurysmal disease to help eliminate the confounding aortic dissection may introduce into the analysis.

The data presented should be taken in the context of varied transfusion practices driven by standard laboratory data, viscoelastic studies, and institutional protocols. Cognizant of this, we utilized this multicenter database and adjusted for center-level differences in our mixed-effect model. Our aim was to evaluate if MHCA could decrease transfusion requirements, a known, frequently cited concern in aortic arch surgery, across a variety of practice patterns. While physiologic coagulopathy associated with hypothermia is hard to refute, its contribution to transfusion requirements may appear smaller than previously appreciated.

### Limitations

Our study has several limitations. First, data were collected retrospectively and may be subject to unmeasured confounding. Second, while limiting our cohort to aneurysmal patients undergoing hemiarch repair improves internal validity, it limits generalizability. Third, we do not have viscoelastic data that have been cited to reduce transfusion requirements. 25 Finally, some patients received platelet dose packs but were not included in the platelet transfusion multivariable analysis.

## Conclusion

MHCA was associated with a minor reduction in FFP transfusion requirements after aortic hemiarch repair for nondissected, aneurysmal disease. However, the impact of temperature during HCA on transfusion requirements in modern cardiac surgical practices appears to be modest; in contrast, CPB remains an important contributor. Shorter CPB times associated with MHCA may facilitate decreased transfusion requirements, but hypothermia-induced coagulopathy may not play as significant of a role as previously thought.
